# Range expansion of the economically important Asiatic blue tick, *Rhipicephalus microplus*, in South Africa

**DOI:** 10.4102/jsava.v88i0.1482

**Published:** 2017-12-08

**Authors:** Nkululeko Nyangiwe, Ivan G. Horak, Luther van der Mescht, Sonja Matthee

**Affiliations:** 1 Department of Rural Development and Agrarian Reform, Döhne Agricultural Development Institute, South Africa; 2 Department of Conservation Ecology and Entomology, Stellenbosch University, South Africa; 3 Department of Veterinary Tropical Diseases, University of Pretoria, South Africa

## Abstract

The Asiatic blue tick, *Rhipicephalus microplus*, a known vector of bovine babesiosis and bovine anaplasmosis, is of great concern in the cattle industry. For this reason, detailed knowledge of the distribution of *R. microplus* is vital. Currently, *R. microplus* is believed to be associated mainly with the northern and eastern Savanna and Grassland vegetation in South Africa. The objective of the study was to record the distribution of *R. microplus*, and the related endemic *Rhipicephalus decoloratus*, in the central-western region of South Africa that comprises Albany Thicket, Fynbos and Savanna vegetation. In this survey, ticks were collected from 415 cattle in four provinces (Eastern Cape, Northern Cape and Western Cape and Free State provinces) and from the vegetation in the Eastern Cape province of South Africa between October 2013 and September 2015. More than 8000 ticks were collected from cattle at 80 localities of which *R. microplus* was present at 64 localities and *R. decoloratus* at 47 localities. A total of 7969 tick larvae were recorded from the vegetation at 20 localities of which 6593 were *R. microplus* and 1131 were *R. decoloratus. Rhipicephalus microplus* was recorded in each of the regions that were sampled. *Rhipicephalus microplus* is now present throughout the coastal region of the Eastern Cape province and at multiple localities in the north-eastern region of the Northern Cape province. It was also recorded in the western region of the Western Cape province and one record was made for the Free State province. The observed range changes may be facilitated by the combined effects of environmental adaptability by the tick and the movement of host animals.

## Introduction

It is well established that ticks and tick-borne diseases (TBDs) significantly impact domestic animal health and the livestock farming industry globally (Busch et al. 2014; De Castro 1997; Jonsson & Piper 2007). In Africa, it is estimated that animal losses because of high tick infestations and the control of TBDs such as babesiosis and anaplasmosis cost countries such as Kenya, Tanzania and Zimbabwe between $5 and $6 million per annum (McLeod & Kristjanson 1999). South Africa has a large commercial cattle farming industry and spends considerably more on TBDs per annum (approximately $21 million) (McLeod & Kristjanson 1999). There are two tick species that act as vectors of the causative agents of bovine babesiosis in South Africa: *Rhipicephalus decoloratus* (the African blue tick), which is endemic to Africa and transmits *Babesia bigemina* (African redwater) and *R. microplus* (the Asiatic blue tick), which is originally from southern Asia and acts as a vector for *B. bigemina* and *B. bovis*, the causative organism of Asiatic redwater in cattle. *Rhipicehalus microplus* is of greater concern in the cattle industry mainly because of the greater pathogenicity of *B. bovis* (De Vos, De Waal & Jackson 2004).

*Rhipicephalus microplus*, originally a parasite of bovid species in India and Indonesia (Barré & Uilenberg 2010; Labruna et al. 2009; Osterkamp et al. 1999), is presumed to have been introduced into Africa via Madagascar during the latter half of the 19th century (Hoogstraal 1956; Madder et al. 2011). The tick has subsequently spread across southern, eastern and western Africa and to date the affected countries include South Africa (Howard 1908; Tønnesen et al. 2004), Zimbabwe (Mason & Norval 1980), Swaziland (Weddernburn et al. 1991), Zambia (Berkvens et al. 1998), Ivory Coast and Benin (De Clercq et al. 2012; Madder et al. 2007), Tanzania (Lynen et al. 2008), Mozambique (Horak et al. 2009), Burkina Faso, Mali and Togo (Adakal et al. 2013) and Namibia (Nyangiwe et al. 2013b).

More specifically in South Africa, Howard (1908) was the first to record *R. microplus* among ticks collected at King William’s Town in the Eastern Cape province (ECP). Thereafter, Howell, Walker and Nevill (1978) recorded *R. microplus* in isolated pockets along the southern coast of the Western Cape province (WCP) in the districts of Humansdorp, Knysna, George, Mossel Bay, Heidelberg, Swellendam and at a few inland localities. Since then, *R. microplus* has successfully become established in the mesic Grassland and Savanna interior regions and is now widely distributed throughout the provinces of Limpopo, Mpumalanga, North West, Gauteng and KwaZulu-Natal (Baker et al. 1989; Spickett, Heyne & Williams 2011; Tønnesen et al. 2004; Walker et al. 2003). However, it is uncertain what the full extent of the tick’s distribution is in the remaining four provinces (Free State, Eastern Cape, Northern Cape and Western Cape provinces). The vegetation in the ECP broadly comprises Grassland (mountain and coastal) and Albany Thicket with Grassland vegetation predominating in the eastern region of the province. The region around East London and King William’s Town forms a transition zone between Grassland and Albany Thicket vegetation, with the latter found mainly in the western region of the province (Mucina & Rutherford 2006). Nyangiwe, Harrison and Horak (2013a) focused their survey on the eastern Grassland region of the ECP (east of East London) and not only found *R. microplus* in high abundance but also provided evidence that *R. microplus* was displacing the endemic *R. decoloratus* and demonstrated the existence of larvae that share morphological features with *R. microplus* and *R. decoloratus* (i.e. suspected hybrids). However, to date it is uncertain whether and how widely *R. microplus* is distributed in the predominant Albany Thicket vegetation. Recent studies suggest that *R. microplus* might be expanding its range further westwards, across South Africa, with isolated records in the western region of the ECP (four localities) (Nyangiwe et al. 2013a), the north-eastern region of the North West province (NWP) (14 localities) (Spickett et al. 2011) and the north-eastern region of the Free State province (FSP) (three localities) (Horak et al. 2015). Evidence of *R. microplus* on wild antelope (Horak et al. 2015; Tonetti et al. 2009) has suggested that the tick is adapting to novel hosts, which will aid in the spread of the tick across South Africa.

In general, current distribution maps for the tick species in South Africa are in need of revision. This is mainly because of the fact that most of the locality data for ticks are either based on historic data (Spickett 2013; Walker, Keirans & Horak 2000) or biased towards a few tick and host species that are of economic importance (Horak et al. 2009, 2015; Marufu et al. 2011; Nyangiwe et al. 2013a). Furthermore, there are several factors such as climate change (Léger et al. 2013; Tabachnick 2010), uncontrolled movement of domestic animals and wildlife (Biello 2011; Bigalke 1994; Fayer 2000; Mackenzie & Norval 1980; Peter et al. 1998), development of acaracide resistance (Mekonnen et al. 2002, 2003) and a recent expansion in host range (i.e. the number and type of host species that are used by ticks) (Horak et al. 2015; Junker, Horak & Penzhorn 2015) that make it possible for ticks to survive and then become established in novel localities. More pertinent to the distribution of *R. microplus* is a possible sampling bias towards mesic Savanna (grasses and trees) and Grassland (predominantly grass layer) vegetation because of the perception that the tick does not occur in drier regions and/or in predominantly woodland and shrub vegetation. This study was conducted in an attempt to address the paucity of information regarding the geographic distribution of *R. microplus* and the related endemic *R. decoloratus*, largely in the Eastern Cape, Northern Cape and Western Cape provinces of South Africa.

## Research method and design

In this study, two methods were mainly used to obtain ticks: (1) active tick removal from cattle (by the authors N.N. and S.M.) and (2) sampling of ticks on vegetation using drag sampling (by N.N.) (Nyangiwe et al. 2011; Spickett et al. 1992). However, in addition, samples (*n* = 5) were also provided by private cattle farmers in response to radio interviews, articles in newspapers and popular magazines.

**FIGURE 1:**
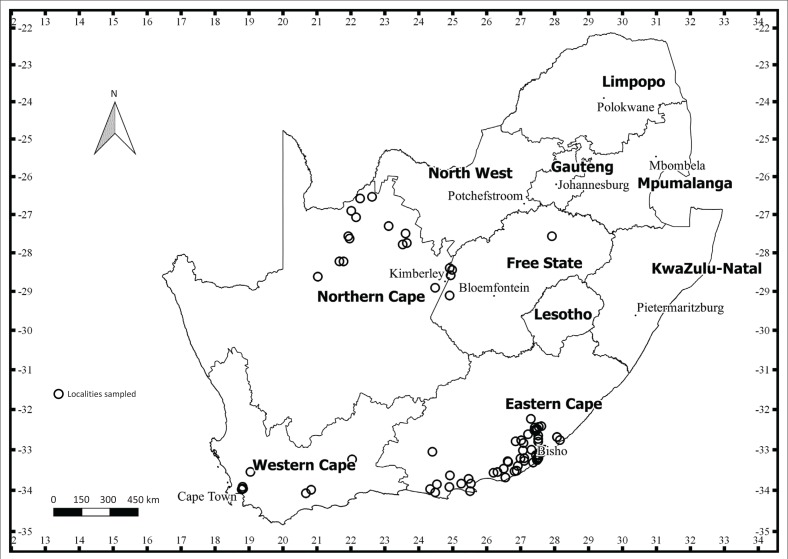
Sampling localities where ticks were collected in the Eastern Cape, Northern Cape, Western Cape and Free State provinces in South Africa (2013–2015).

Ticks were collected from cattle on farms in the region west of East London in the ECP, in the north-eastern region of the Northern Cape province (NCP), mainly the south-western region of the WCP and at one locality in the north-eastern region of the FSP between October 2013 and March 2015 (Figure 1). The vegetation in the ECP where sampling was conducted is classified as Albany Coastal Belt (close to the coast with short grasslands and bush clumps), Amathole Montane Grassland (short Grassland near mountains with undulating slopes), Bhisho Thornveld (open Savanna characterised by small trees) and Great Fish Thicket (woody trees, shrubs and the succulent component are well developed) (Mucina & Rutherford 2006). The vegetation in the WCP primarily comprises low to medium shrub-like vegetation that represents two vegetation types: Fynbos and Renosterveld (Mucina & Rutherford 2006). The vegetation in the north-eastern region of the NCP is classified as Kalahari Bushveld. In this region, sandy dunes are covered with shrubs, grasses and some trees (Mucina & Rutherford 2006).

At each locality, 3–6 cattle were examined for ticks. Attention was paid to the predilection sites of blue ticks and the ears, neck and dewlap, abdomen, feet, tail and perianal region of each animal were carefully examined (Baker & Ducasse 1967). As the survey was aimed at determining the geographic distribution of *R. microplus* and *R. decoloratus* and not their prevalence or intensity of infestation, none of the collections that were made from cattle were intended to be complete. The ticks from each animal were preserved separately in labelled sample bottles containing 70% ethanol. Information on date, farm, breed, sex and age of the host was recorded. In addition to on-host tick collections, ticks were collected from the vegetation at 20 localities in the ECP (five in each of the four above-mentioned vegetation types) using drag sampling during October 2012–February 2013. The drag sampling method is described in detail in Nyangiwe et al. (2013a). The geographic coordinates of each locality were recorded. All the ticks were identified to species level and counted using a Leica stereoscopic microscope (Leica Microsystems, Wetzlar, Germany) and the taxonomic key of Walker et al. (2003). Species identification was confirmed by I.G. Horak. The geographic coordinates were used to plot the distribution of the two tick species in QGIS v 2.6.1 (Quantum GIS Development Team 2015).

## Results

A total of 8408 adult ticks were collected from cattle from 80 localities in the ECP, WCP, NCP and FSP. Of the ticks, 6034 (71.8%) were identified as *R. microplus* and 2374 (28.2%) as *R. decoloratus*. Overall, the two species were sympatric at 40 (50%) localities, with *R. microplus* present at more localities (80%) than *R. decoloratus* (58.8%) (Table 1).

**TABLE 1:** Occurrence data for *Rhipicephalus microplus* and *Rhipicephalus decoloratus* from cattle in the Eastern Cape, Northern Cape, Western Cape and Free State provinces in South Africa during 2013–2015.

Province	Nloc	Nanimal	Total ticks	Loc *R. microplus*		Loc *R. decoloratus*		Sympatric	
				*n*	%	*n*	%	*n*	%
Eastern Cape	53	318	8101	51	96.2	33	62.3	32	60.4
Northern Cape	18	64	72	8	44.4	10	55.6	5	27.8
Western Cape	8	28	226	4	50	3	37.5	2	25
Free State	1	5	9	1	100	1	100	1	100
**Total**	**80**	**415**	**8408**	**64**	**80**	**47**	**58.8**	**40**	**50**

Nloc, number of localities sampled; Nanimal, number of cattle examined; Loc *R. microplus*, localities positive for *Rhipicephalus microplus*; Loc *R. decoloratus*, localities positive for *Rhipicephalus decoloratus*; Sympatric, localities where species co-occurred.

**FIGURE 2:**
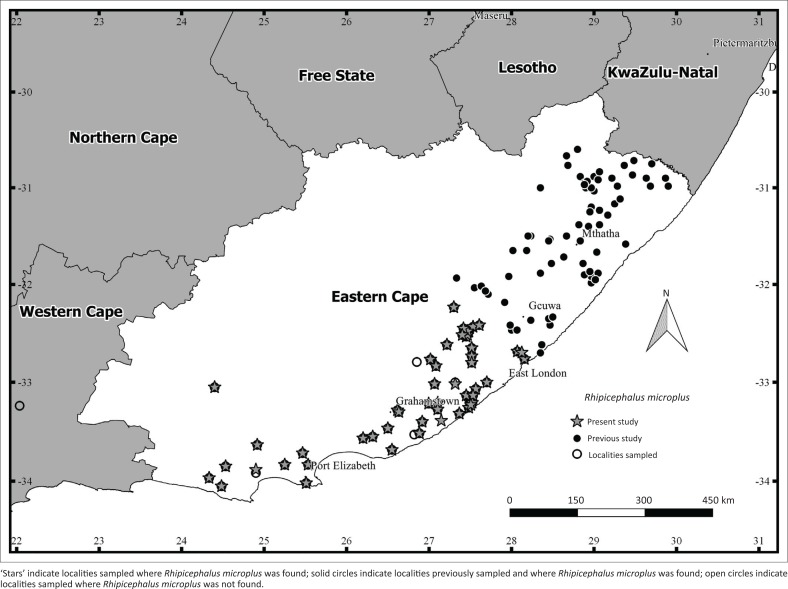
Sampling localities where ticks were collected and localities positive for *Rhipicephalus microplus* in the Eastern Cape province in South Africa (2013–2015).

**FIGURE 3:**
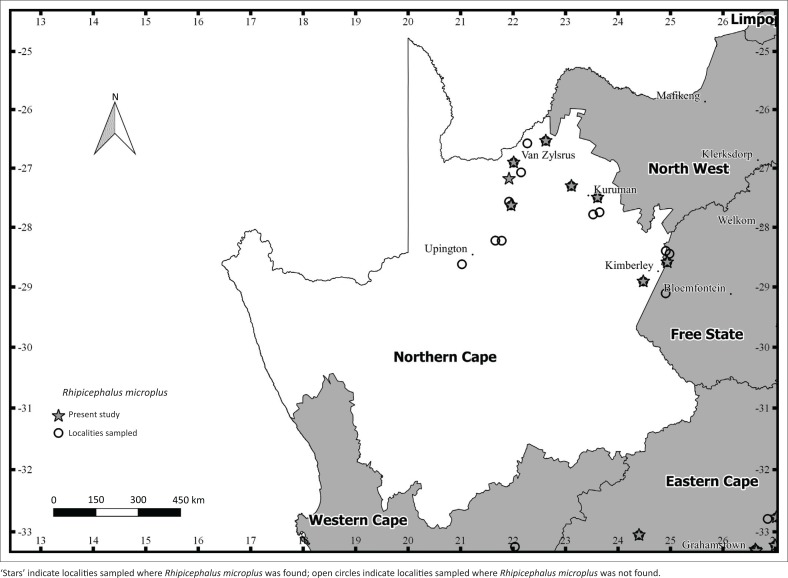
Sampling localities where ticks were collected and localities positive for *Rhipicephalus microplus* in the Northern Cape province in South Africa (2013–2015).

In the ECP, *R. microplus* was recorded from cattle at more localities (*n* = 51) compared to *R. decoloratus* (*n* = 33). In particular, *R. microplus* was recorded at most of the sampling localities (51 of 53) in the predominantly Albany Thicket vegetation in the western region of the ECP (Figure 2). In addition, larval stages of both species and larvae exhibiting characteristics of both species were collected from the vegetation in the western region of the ECP (Table 2). The abundance of *R. microplus* larvae was higher (6593) than that of *R. decoloratus* larvae (1131) and larvae suspected of being hybrids were recorded at each of the 20 sampling localities (Table 2). In the NCP, 18 localities were sampled and *R. microplus* was recorded on cattle at fewer localities (*n* = 8) than *R. decoloratus* (*n* = 10). *Rhipicephalus microplus* was specifically recorded in the furthest north-eastern region of the province close to Van Zylsrus, Kuruman and Kimberley (Figure 3). *Rhipicephalus decoloratus* shared this distribution but was recorded additionally at two localities closer to Upington (map not shown). In the WCP, *R. microplus* was found on cattle at more localities (*n* = 4) than *R. decoloratus* (*n* = 3). Specifically, *R. microplus* was recorded close to Kuilsriver, Wellington and Swellendam (map not shown). Ticks were obtained from cattle at a single locality (close to Heilbron) in the FSP (map not shown) and both tick species were recorded (Table 1).

**TABLE 2:** Total number of *Rhipicephalus microplus, Rhipicephalus decoloratus* and *Rhipicephalus decoloratus*–*Rhipicephalus microplus* suspected hybrid larvae collected from vegetation at 20 communal areas in the Eastern Cape province of South Africa.

Vegetation type	Locality	*Rhipicephalus microplus*	*Rhipicephalus decoloratus*	Hybrids	Total
ACB	Bhola	301	16	9	326
	Dowu	395	68	1	464
	Mazikhanye	346	17	18	381
	Pozi	192	23	3	218
	Tyhusha	268	62	6	336
AMG	Hekele	306	1	12	319
	KwaZidenge	609	6	28	643
	Mgwali	371	5	21	397
	Ndakana	479	15	7	501
	Toyise	712	14	12	738
BT	Dontsa	376	3	7	386
	Madubela	314	138	18	470
	Majali	277	79	6	362
	Lusasa	43	13	6	62
	Sotho	173	276	2	451
GFT	Gcinisa	394	19	20	433
	Mkhanyeni	244	5	8	257
	Peddie Extension	218	7	35	260
	Pikoli	51	344	12	407
	Upper Mgwalana	524	20	14	558
					
**Total**	**-**	**6593**	**1131**	**245**	**7969**

ACB, Albany Coastal Belt; AMG, Amathole Montane Grassland; BT, Bhisho Thornveld; GFT, Great Fish Thicket.

## Ethical considerations

Permission to conduct this study was granted by Stellenbosch University Ethics Committee for Social Sciences (reference no. DESC_Nyangiwe 2012).

## Discussion

The study provided evidence that *R. microplus* is widely distributed along the western region of the ECP and has established its presence in the north-eastern region of the NCP.

In the ECP, *R. microplus* was commonly found on cattle and vegetation (51 and 20 localities, respectively) in the region west of east London. In addition, *R. microplus* was present on cattle at more localities than *R. decoloratus* (51 and 33 localities positive, respectively) and in higher abundance on the vegetation (6593 and 1131, respectively). Albany Thicket vegetation is dominated by shrubs and succulents, although several C_3_ and C_4_ grass species are also present (Mucina & Rutherford 2006). Although the vegetation in the Albany Thicket differs from Grassland vegetation in terms of plant diversity, it appears to provide equally good forage for cattle, as several communal cattle farming areas and large numbers of cattle are found in the region. These factors may explain the presence of both blue tick species in the western regions of the ECP. An earlier study by Nyangiwe et al. (2013a) recorded *R. microplus* at four localities (Majali, Ncerha, Pumprock and Shweme) in the communal grazing areas in the western region of the ECP. The present study therefore provides evidence for further expansion of the geographic range of *R. microplus* in this region, as the tick was recorded at 51 novel localities. Prior to Nyangiwe et al. (2013a) and the present study, the tick was reportedly absent in the region (Mekonnen et al. 2002, 2003; Rechav 1982). Counts of tick larvae from vegetation can give us partial data on abundances of the two species. As mentioned above, the overall abundance of *R. microplus* larvae was higher than that of *R. decoloratus*. This pattern was recorded at 18 of the 20 localities where ticks were collected from the vegetation. The presence of suspected hybrid larvae on the vegetation at all 20 localities supports previous studies that noted that male *R. microplus* attach to and possibly mate with female *R. decoloratus* (Londt & Arthur 1975; Tønnesen et al. 2004). Nyangiwe et al. (2013a) recorded 17 such couplings on cattle and also reported suspected hybrid larvae (*R. microplus x R. decoloratus*) on the vegetation at two communal areas in the ECP.

This study provides the first record of *R. microplus* in the NCP. The fact that *R. microplus* was present at only some of the localities (8 of 18) that were sampled suggests that its presence in the NCP reflects a recent introduction. This hypothesis is supported by the fact that the localities that were positive for *R. microplus* were situated close to the border with NWP (near the towns of Kimberley, Kuruman and Van Zylsrus). Spickett et al. (2011) recorded *R. microplus* in the eastern regions of NWP, while *R. decoloratus* was widespread. The western region of NWP and the north-eastern region of the NCP have comparable vegetation (Kalahari Bushveld and Central Bushveld vegetation, respectively) and climatic conditions (Mucina & Rutherford 2006; Winterbach et al. 2000) and it is therefore possible that *R. microplus* could have recently moved with infested animals across and between provinces.

The indigenous vegetation in the WCP is mainly shrub-like Fynbos and contains little grass (Mucina & Rutherford 2006). Consequently, cattle farmers often irrigate pastures to provide feed for cattle. In the present study, ticks were collected from cattle in the south-western region of the WCP. *Rhipicephalus microplus* was recorded at four of the eight localities. The farms that were positive for *R. microplus* and *R. decoloratus* all had irrigated pastures. This confirms earlier reports that blue ticks prefer grass over other vegetation types and it also suggests that *R. microplus* might have a more patchy distribution across the shrub-dominated WCP when compared to provinces that have predominantly Savanna and Grassland vegetation (Howell et al. 1978; Walker et al. 2003). The presence of *R. microplus* in the WCP may again be because of the movement of cattle within the province and across the country. The cattle in Wellington that were positive for *R. microplus* are part of a breeding stud, and animals are regularly transported between Wellington and the northern Grassland or Savanna regions of South Africa. It is thus possible that the cattle became infested with *R. microplus* during one of the visits to the Grassland or Savanna regions and the ticks subsequently returned with the cattle to the particular farm. One farmer in the Cape Flats or Kuilsriver area reported that he had purchased animals from the ECP and subsequently recorded calf deaths, which were confirmed to be caused by *B. bovis* infection. All of the localities in the Stellenbosch region were negative for *R. microplus* at the time of the survey. However, subsequently one of the farmers recorded cattle deaths, which were confirmed to be caused by *B. bovis* infection. This farmer regularly sources cattle from local farms and must have acquired the tick through cattle movement within the province.

Although this study can only report on ticks recorded at one locality in the FSP (Heilbron), it is the fourth study that recorded *R. microplus* (three female and three male ticks) in this province. Previous studies recorded low numbers of *R. microplus* from cattle at localities close to the border of already infected provinces, for example, in the north-western region (Hoopstad) close to the border of NWP (Tonetti et al. 2009), in the south-eastern region (south of Harrismith) close to the border with KwaZulu-Natal (Spickett 2013) and in the north-eastern region (north of Heilbron) close to Gauteng and in the south-eastern region (north of Clarens) close to the border with KwaZulu-Natal (Horak et al. 2015).

## Conclusion

This study provides evidence that *R. micoplus* has increased its distribution range in South Africa and can now be found throughout the Albany Thicket vegetation of the ECP, in the Bushveld vegetation of the NCP and in isolated patches in the Fynbos vegetation of the WCP. It is predicted that the establishment of the Asiatic blue tick in naïve environments will result in higher disease incidence and tick-related deaths. This is supported by the fact that several *B. bovis*-related deaths have recently been recorded among naïve cattle in the WCP.
